# 3D printing approaches to simulate natural diets for insects with implications for domestication and mass-rearing

**DOI:** 10.3389/finsc.2025.1674092

**Published:** 2025-11-19

**Authors:** Carlos Pascacio-Villafán, Viridiana Tejada-Ortigoza, Allen Carson Cohen, Martín Aluja

**Affiliations:** 1 Red de Manejo Biorracional de Plagas y Vectores, Clúster Científico y Tecnológico BioMimic^®^, Instituto de Ecología, A.C., Xalapa, Veracruz, Mexico; 2 School of Engineering and Sciences, Tecnologico de Monterrey, Monterrey, Nuevo León, Mexico; 3 Department of Entomology and Plant Pathology, Insect Rearing Education and Research, North Carolina State University, Raleigh, NC, United States

**Keywords:** 3D printing, state-of-the-art technology, diet development, customized diet, domestication, sterile insect technique, insect rearing

## Abstract

Many insect species that could benefit humanity cannot be reared or domesticated because of the lack of adequate artificial diets. In the case of insect pests which are controlled via the Sterile Insect Technique, the prospects of improving biological traits of mass-reared individuals hinge on the development of new diet formulations. 3D food printing technology holds unique potential to create customized artificial diets simulating the shape, texture, and distribution of nutrient and non-nutrient components (e.g., dietary fiber and secondary metabolites) of natural insect foods. We present an overview on the use of state-of-the-art 3D printing technology to develop artificial diets that mimic the dynamic nature of natural insect diets, characterized by compartmentalized food components. The challenges and limitations of 3D food printing technology for its application in the field of diet development and artificial insect rearing are discussed, and future research priorities are highlighted. Examples are provided of beneficial and pestiferous insect species that could be reared on 3D-printed diets such as the cocoa pod borer and tephritid flies.

## 1 Introduction

Human control and use of insect populations has a long history starting with the domestication of silkworms [*Bombyx* spp. (Lepidoptera: Bombycidae)] and honeybees (*Apis* spp. (Hymenoptera: Apidae)) thousands of years ago (1). Today, trillions of insects are produced annually in many farms, factories and laboratories around the world to obtain fiber, food and feed products, or for crop pollination, pest control, waste management services, and research, to mention but a few benefits of captive insect populations (2–6).

A crucial factor in the domestication and management of insects for the above-mentioned purposes is the provision of high-quality feeds or diets of the insects being colonized, domesticated and mass-reared (5, 7). Diets for insect rearing can be natural or artificial (2). An example of a natural diet are the mulberry leaves, *Morus* spp. L. (Rosales: Moraceae), used for rearing silkworms that produce ca. 90% of the world’s total raw silk (8, 9). On the other hand, artificial diets are human-made foods synthesized from ingredients with varying levels of chemical purity such as the holidic (i.e., chemically defined) diet formulation for laboratory rearing of the vinegar fly, *Drosophila melanogaster* Meigen (Drosophilidae) (10), the most widely used insect model organism in biological and medical research (6). Despite advances in the science and technology of insect rearing, a major barrier to our ability to domesticate and establish managed populations (colonies) of many insect species is the lack of adequate artificial diets (2).

Here, we propose adoption of 3D (three-dimensional) printing technology for development of novel insect diets, as a way of better imitating the natural foods of insects. 3D printing is considered a general purpose and revolutionary technology with several applications in many fields (11–14), but its use in the design and development of artificial diets for insect rearing remains largely unexplored. We present an overview of 3D printing and introduce several examples of the uses and innovations that this technology promises in studies with insects. The principles and techniques of 3D food printing that could be applied to configure artificial diets that simulate the natural compartmentalization of insects’ foods are described. The relationship between insect domestication and insect diets is discussed, highlighting the unique potential of 3D food printing technology to simulate the dynamics of the spatial organization of natural insect foods. Some aspects related to the feeding biology of insects of economic, ecological and medical importance, relevant to the development of artificial diets, are described. We finish by outlining future research and perspectives that we believe should be addressed to facilitate the use of 3D food printing technology for the development of 3D-printed artificial diets for inset rearing.

## 2 Overview of 3D printing technology and its use in studies with insects

3D printing technology, formally known as “additive manufacturing”, had its origins in the 1980s with the work of Kodama (15) and a patent by Hull (16). It is an automated manufacturing method characterized by the layer-by-layer deposition of a particular material following a pre-designed digital 3D model (17, 18). Models can be generated by scanning the actual objects to be printed, by computer-aided design (CAD) software, or downloaded from online sources (13, 19). One of the main advantages of 3D printing is customization or personalization, allowing the creation of objects adapted to specific needs and that could not be obtained with other methods (13).

3D printing technology has induced innovations in many fields of research with insects including the development of artificial nests printed with biodegradable material to support wild cavity-nesting bee populations (20), synthetic beetle decoys for capturing invasive forests pests (21), bioinspired designs of artificial insect wings to study the mechanics of insect flight (22), prints of eggs of invasive insect species and of defoliated soy bean leaves for pest management education (23), and prints of cost-effective feeders for laboratory assays with mosquitoes vectoring malaria (24, 25). Overall, 3D printing technology allows the development of realistic models of various organisms or parts of organisms such as flowers to study the feeding response of moths to flower morphology (26), fruits to study predation of cryptic prey insect species (27), and insect models to disseminate information about museum specimens (28). Special rearing trays and containers for rearing insects for food and feed purposes (29) and for research (30), have also been created with 3D printing technology.

The above examples make it clear how 3D printing technology has contributed to the development of research on insect biology, ecology, conservation, pest management, among other areas of insect science. However, other fundamental areas such as artificial diet development have not adopted this technology. This is unfortunate because the overall progress of entomology is directly related to our ability to rear and produce insects on artificial diets (2, 3).

## 3 3D food printing

3D food printing was first used to manufacture cakes, cheese, and cookies (31, 32). In the last decade, 3D food printing technology has allowed the development of personalized meals designed to cover consumers’ specific nutritional and sensory requirements (14, 33). 3D printing customized foods was key to the European-funded project PERFORMANCE (Personalized Food for the Nutrition of Elderly Consumers), which was focused on elderly people with dysphagia (i.e., difficulty in swallowing), nutritional deficiencies, or that were malnourished (34, 35). In this regard, food that might not be visually appealing, because it is a paste-like formulation, might become healthy, tasty and attractive to consumers through 3D printing. For example, it is possible to print “beef” steaks with varying levels of intramuscular fat and tenderness, among other organoleptic characteristics that define the quality of a steak, using plant-based ingredients (36, 37). Furthermore, some companies use 3D printing technology to manufacture plant-based alternatives to meat and fish (e.g., 38). The 3D printing of meat and other complex food formulations allows the development of customized food with a high level of similarity to the texture, smell and flavor of the original food (39, 40). On top of allowing the creation of complex, visually appealing and nutritious foods, 3D printing improves food manufacturing by reducing food-related waste and accelerating supply chains (18).

To achieve the desired textures and nutritional values in 3D-printed foods, it is essential to consider the rheological, thermal, and mechanical characteristics of the printing materials (18, 41). For instance, characteristics such as particle size and the bonding strength of materials influence the structural integrity of 3D-printed food products (18). Major barriers for the broad adoption of 3D food printing include the high cost of printing equipment and materials, and regulatory issues such as food safety and labeling (18).

## 4 Techniques and principles of 3D food printing

**Figure 1:**
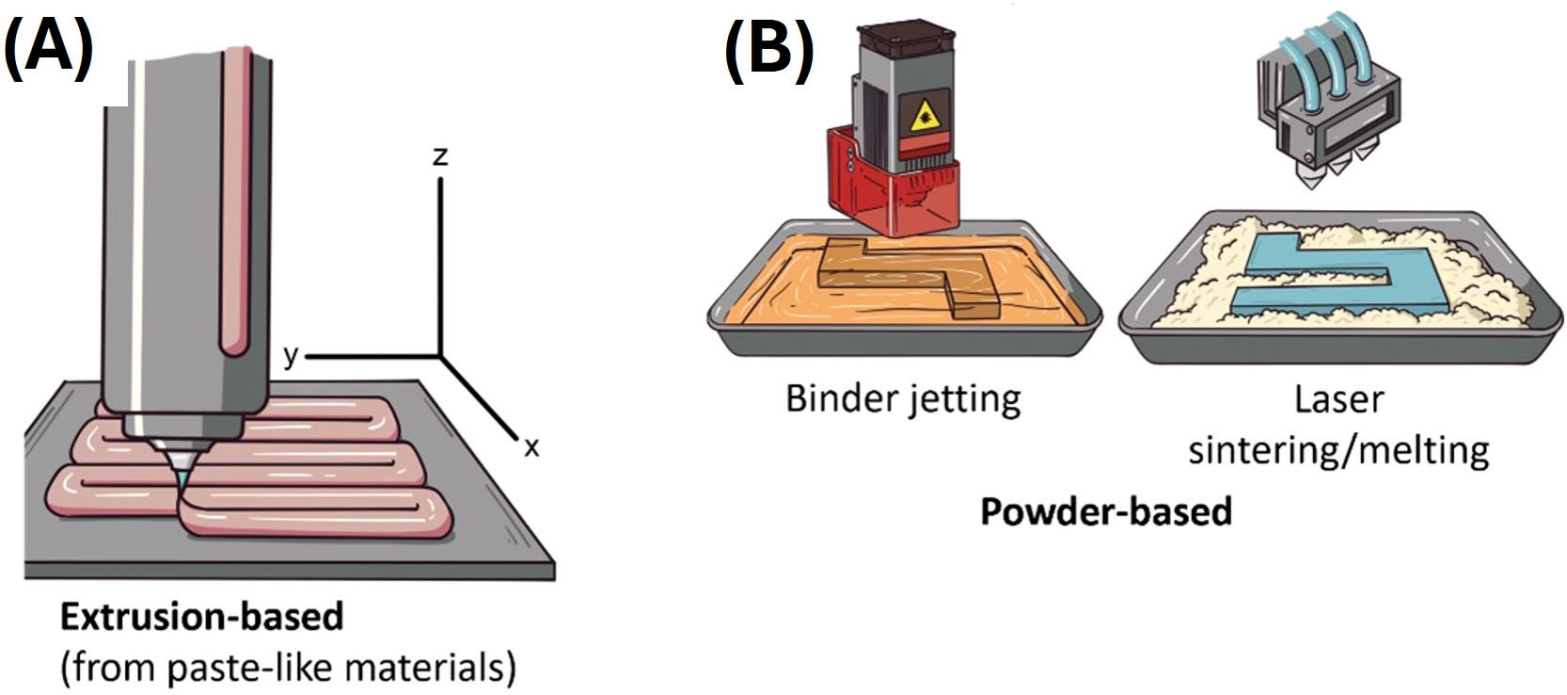
Depiction of 3D food printing techniques. **(A)** Extrusion-based and **(B)** powder-based 3D food printing techniques.

The better-known techniques in food 3D printing are extrusion, binder jetting and selective laser sintering (**Figures 1A, B**), with extrusion being the most used (a detailed analysis of the different 3D food printing technologies can be found in Sohel et al. (18)). The extruder is equipped with a three-degrees of freedom mechanism, allowing it to move across three spatial dimensions (x, y, and z axis, **Figure 1A**). The process can occur in a range of temperatures depending on the characteristics of the printing materials (41). Some materials require heating to be melted or semi-melted for their extrusion (41). Pseudoplastic materials (i.e., paste-like materials under certain conditions) are ideal because, commonly, they flow through the nozzle (i.e., the part of a 3D printer that deposits filaments of the printing material) once a force is applied, and they hold their structure and dimensions once they have been extruded and deposited (42). These are key characteristics that materials (or formulations) must have for extrusion printing (42). The processing parameters for extrusion-based techniques are highly dependent on the printer brand and model, but controllable printing variables include nozzle diameter, nozzle moving speed, printing temperature, layer height, and extrusion rate (43, 44). The thickness of the filaments printed by extrusion using food materials can range from µm to mm (45). Several printing materials such as meat (36, 46), vegetables (47–49), surimi (50), cereals (51–53), fruits (54), insect meals (55), among many others, have been used for 3D printing by extrusion. Hydrocolloids are key additives for improving consistency and structural integrity post-printing in 3D-printed food products (56). Ingredients such as sodium alginate, xanthan gum, guar gum, agar and gelatin, add viscosity and texture to 3D-printed foods (56).

Powder-based techniques (binder jetting and selective laser sintering, **Figure 1B**) consist of a powder material bed that is refilled continually. These techniques depend on the chemical and/or physical linkage or reaction of the powder with specific agents (18). For instance, binder jetting works with a liquid binder that solidifies the powder; the printer mechanism manages the dropping of the binder in the powder bed (18, 41). Cellulose and xanthan gum have been used for printing food through binder jetting (57). For selective laser sintering, the printer carries a laser source (this can also be a hot air beam at high temperatures) that performs the fusion or melting of the powder (18, 41). Ingredients like maltodextrin and starch as powders, and gluten, glucose and maltodextrin as binders, were used for selective laser sintering food printing (58). Powder-based techniques are convenient for printing complicated structures (18). However, printing complex formulations (several ingredients) with these techniques can be difficult compared to extrusion, due to the chemical composition and interaction of the macromolecules of each ingredient (42).

## 5 Insect domestication and the potential use of 3D food printing to improve artificial rearing

Domestication is considered a coevolutionary process based on a mutualistic relationship in which one species constructs a niche suitable to manage the survival and reproduction of another species, from which it obtains resources and other benefits (59). Insect domestication has a history of ca. 5,000 years starting with silkworms (1). The initial steps in insect domestication includes the acclimatization and colonization of a wild population to human-controlled environments, followed by different levels of control of its life cycle and the eventual development of selective breeding programs and improved strains (7, 60, 61). For example, the domestication of silkworms started with wild individuals of an extant wild silk moth species (*Bombix mandarina* (Moore)), but nowadays there are thousands of strains of highly silk-productive individuals of *Bombix mori* L. adapted to specific farming environments and that could not survive and reproduce without human assistance (3, 7, 62). Recent domestication programs of species such as the black soldier fly, *Hermetia illucens* L. (Stratiomyidae), and the yellow mealworm, *Tenebrio molitor* L. (Tenebrionidae), used in the feed and food industries, aim to develop strains resistant to extreme temperatures and extreme population densities (62). Other domesticated insect species include crickets, such as a white-eyed inbred strain of *Gryllus bimaculatus* (De Geer) (Gryllidae), which are produced for food applications, teaching and scientific research (63); and fruit fly (Diptera: Tephritidae) parasitoids (Hymenoptera) for augmentative biological control (61). The development of genetic sexing strains that allow the separation of sexes during the egg or pupal stage of holometabolous insects and the rearing of only male individuals, has been especially important in fruit fly (Tephritidae) pest species that are mass-produced in factories for Sterile Insect Technique (SIT) applications (3, 64–68).

One key factor for the successful domestication of insects is the provision of diets that allows high growth and survival rates of individuals (3, 5, 69). However, many insect species with potential applications for human needs cannot be reared and maintained in captivity because of specialized feeding adaptations and dietary needs (5, 6). To be a successful component of insect colonization, artificial diets must contain the necessary feeding stimuli for palatability, the required nutrients and bioavailability that mirrors natural foods (2). Artificial diets must reflect the organization of nutrients and non-nutrient compounds found in natural diets to match the feeding adaptations of the insects (2). The feeding adaptations include pin-point feeding capabilities that target specific biomass components such as interstitial cells between vascular bundles, high nutrient cells in fruit pulp or seeds, and various other feeding targets of insects with specialized feeding adaptations (2, 70). The high levels of organization of natural foods and their components are characterized extensively in a compartmentalized matrix or a “dispersion” (*sensu*^2^, ^71^). The dispersion concept advanced by Walstra and von Vliet (2008) is highly complex and has far-reaching implications that are beyond the scope of this article. But suffice it to say that the way components are arranged in relationship to one another has ramifications that explain much of the insects’ responses to their natural and artificial diets (2).

Here, we argue that 3D printing holds the potential beyond any previously attempted technology to simulate the organization of natural foods to match the special feeding adaptations of many species of insects. Such an accomplishment would greatly enhance our ability to rear many recalcitrant insects that have defied previous rearing efforts including hemipteran and dipteran species (72–74). We are optimistic about the potential of 3D printing of insect diets (media or feeds) to provide the chemical and physical (or textural) characteristics of the dispersions discussed by Walstra and von Vliet (71). In the following sections, we explain how engineering diets can be achieved by understanding the dynamics of the spatial organization of living systems/tissues so that 1) palatability (sensory stimulation), 2) nutritional value, 3) bioavailability, and 4) stability of diets can be achieved with 3D printing technology. We apply concepts of organization of existing artificial diets to prospects in using 3D printing to achieve the four properties of superior diets, by exploring the “spatial organization” concept (2, 3).

## 6 Natural diets of insects are dynamic and changing systems with compartmentalized food components

Natural foods of most insects mostly contain living or recently living tissues. Simulation of living food complicates the development of artificial diets in several ways, the most important being the spatial distribution of natural foods and the spatial organization of non-nutrient components (including defensive components such as latex and secondary metabolites in plants). These factors constitute the characteristics of living (or recently living) foods, and it leads to the dynamic nature of changes in living systems. For example, photosynthesizing plants generate free radicals, produce nutrients such as sugars, and undergo a constant flux of synthesis and degradation of compounds such as ascorbic acid, which is continuously being synthesized and destroyed (75–77). The dynamic flux of ascorbic acid and the enzyme that destroys it, ascorbate oxidase, affect signaling systems where ascorbate oxidase is sequestered and released alternately for signaling and coordination of biochemical pathways (75, 76). Plants also present defensive barriers such as mechanical structures (e.g., spines and trichomes) and biochemical signals (78). Also inherent in the organization of plants and other living organisms are synergistic and antagonistic interactions of components such as secretion of cuticles with waxy layers that help maintain water balance and surface resistance to attack by microbes but can also repel or restrict penetration by insects’ mouthparts (79–82). Ascorbic acid is an example of a component of many organisms, which can have beneficial effects where it protects other metabolites from oxidation/deterioration, but it can act as a synergist for iron to become a powerful agent of attack on double bonds of lipids and other biochemical components (2). Another type of interaction that is important in the context of the dynamic and changing nutritional environments of insects, and on which we will delve later in the section on tephritid fruit flies, is that of beneficial microorganisms that colonize the substrates on which the insects feed (e.g., fruit) and that significantly contribute to larval nutrition (83).

### 6.1 Nutrient concentrations and nutritional density (or richness) in leaves and stems

**Figure 2:**
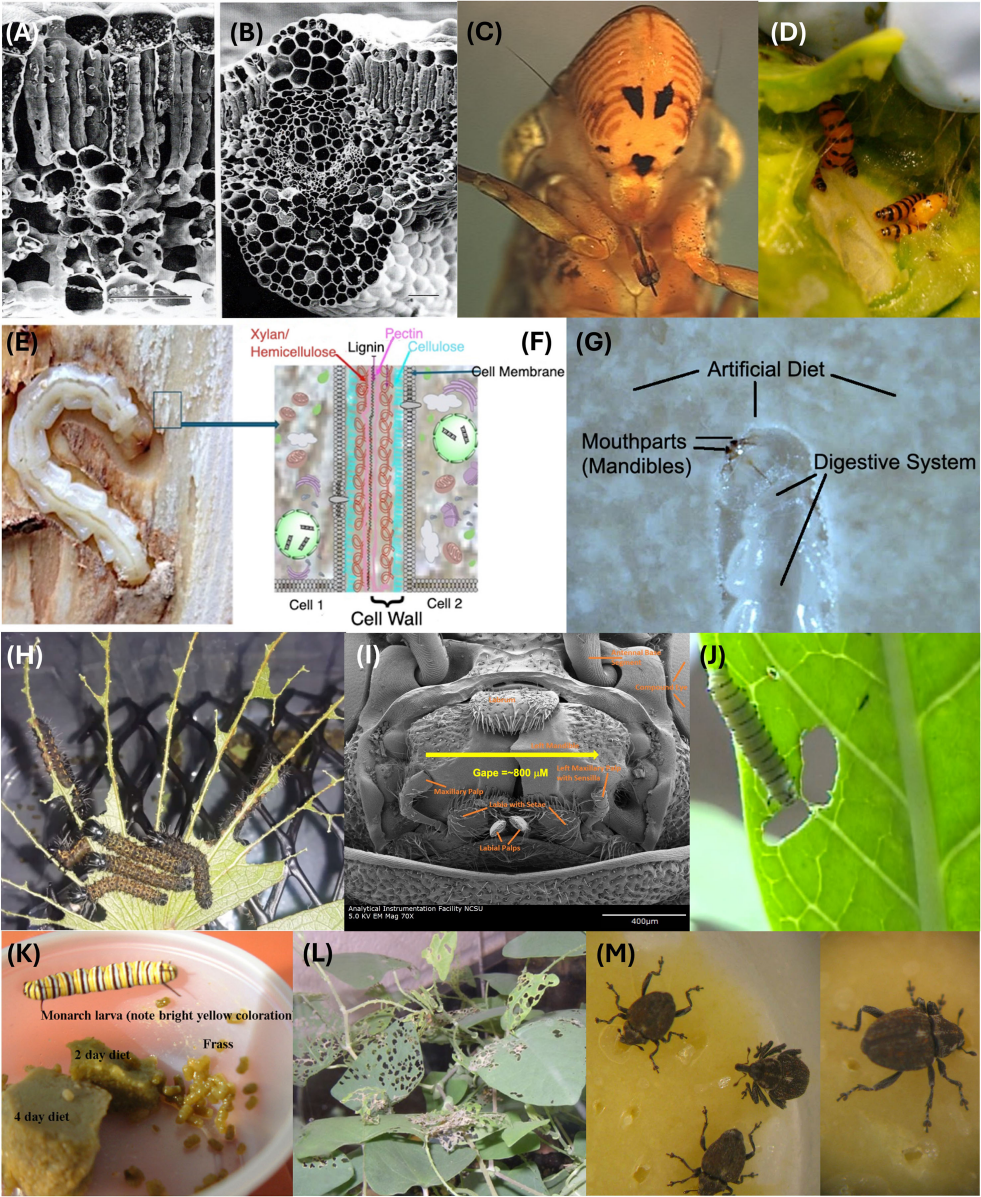
Examples of leaf- and stem-eating insects, compartments and artificial diets designed to mimic the compartments. **(A, B)** Scanning electron micrograph images of cotton leaf, showing elongated palisade cells, spongy mesophyll, and vascular bundles (large xylem cells, smaller phloem elements). Bars in **(A, B)** = 50 µM. Note the cells/tissues that are rich in organelles such as protein-rich chloroplasts. Note further that the vascular bundle cells lack organelles that could provide nutrients for insects lacking digestive systems capable of concentrating dilute saps. **(C)** Bottom view of the head of the glassy-winged sharpshooter (*Homalodisca coagulata*) showing a piercing and sucking mouthpart specially adapted for feeding on low nutrient foods such as xylem sap. **(D)***Opuntia* sp. (prickly pear) cactus infested with *Cactoblastis cactorum* larvae. **(E)** A larva of the emerald ash borer (*Agrilus planipennis*) in its feeding gallery in an ash tree with its bark removed, and **(F)** a magnification and superimposition of the phloem wood cells with their organelles; **(G)** a larva of *A. planipennis* eating a heterogeneous artificial diet made with wheat germ, casein, and cellulose in an agar gel. **(H)** Mopane worms, *Gonimbrasia belina*, consuming mopane (*Colophospermum mopane*) leaf. The larvae select the interstitial tissues, leaving uneaten the leaf veins. **(I)** Scanning electron micrograph of the mouthparts of an adult *A. planipennis*, showing the gape of the mandibles (800 µM). **(J)** Milkweed leaf being eaten by a monarch butterfly larva, *Danaus plexippus*. Note that the larvae, especially early instars, chew laticifers that carry milkweed latex-containing sap to the wounded leaf tissues. This practice reduces the exposure of larvae to gummy, toxin-containing latex, allowing the larva to feed on interstitial leaf tissues that contain reduced amounts of latex. **(K)** Monarch butterfly larva with artificial diets, showing diet deterioration with age, as indicated by the faded green color of the diet slab. **(L)** Leaf feeding damage on *Persicaria perfoliata* from *Rhinoncomimus latipes* adults; and **(M)***R. latipes* adults feeding on an artificial diet designed to mimic the nutritional contents of the *P. perfoliata* leaf. Photos credit: **(A-G)** and **(I-M)** Allen C. Cohen, **(H)** Thabang Pertunia Moropa and Godfrey Mwanza.

Leaves and stems are less nutritious than seeds and fruits (2). This imposes upon insects that consume leaves and leaf-parts and/or stems/stem-parts the necessity of ingesting the most nutrient-rich (high protein and lipid-rich) cells and tissues (2, 70). For example, the palisade cells and spongy mesophyll in leaves (**Figures 2A, B**) contain abundant protein-rich chloroplasts, which also contain phospholipids and vitamins associated with photosynthesis (2, 84). Palisade layer cells and spongy mesophyll in leaves also serve as nutrient reservoirs for storage of starches and lipids (2).

In contrast to moderate nutrient availability in leaves, stems are less nutritious because of their inherent nature of support and vascular transport of components such as water and raw materials for photosynthesis (84, 85). Therefore, insects adapted to utilize or reside in stems tend to be adapted to induce galls (e.g. the eurytomid wasp *Tetramesa romana* Walker, and *R. latipes* larvae). Galls, which are especially rich in proteins and lipids, are a special case of compartmentalization and are unique in being formed by the insect’s manipulation of the plant’s defenses and metabolism (86).

Exceptions to the gall-forming strategy where insects utilize stems, include insects adapted to feed on xylem sap or phloem sap (70). These insects have specialized feeding systems (mouthparts and guts) that are adapted to finding and penetrating xylem bundles or phloem elements, with the guts of these insects being specially adapted to concentrating otherwise scant nutrients in the fluids of plants present in the vascular bundles (70). An example of xylem sap feeders is the glassy-winged sharpshooter, *Homalodisca coagulata* (Say) (Hemiptera: Cicadellidae) (**Figure 2C**). Other low-nutrient specialists where 3D printing technology could be useful for artificial diet development, include the cactus moth larva, *Cactoblastis cactorum* Berg (Pyralidae), and invasive species that threatens native *Opuntia* spp. (Cactaceae) populations in Mexico and USA (**Figure 2D**), and wood-eating insects such as the emerald ash borer larva, *Agrilus planipennis* Fairmaire (Coleoptera: Buprestidae) (**Figures 2E–G**).

## 7 Overview of engineering diets that reflect plant compartments

### 7.1 Leaf eaters

Considering major nutrient classes (i.e., proteins, carbohydrates, lipids, vitamins, and minerals), natural foods of insects are heterogeneously distributed. For example, leaf tissue-feeders, such as the Mopane worm (*Gonimbrasia belina* Westwood (Lepidoptera: Saturniidae)) larvae (**Figure 2H**), an edible species that is an important source of protein for poor people in Southern Africa (87), consume the cellular materials in the epidermis and mesophyll tissues, including palisade layers and spongy mesophyll, which contain the major nutrient groups. Strategies for developing diets for leaf feeders should consider the insect’s mandible morphology and bite gape (**Figure 2I**) so that the thickness and consistency of the diet allows for easy and selective feeding (70, 88).

The monarch butterfly larva, *Danaus plexippus* L. (Nymphalidae) (**Figures 2J, K**), feeding on the milkweed leaf is an example of the micro-feeding dynamics described by Cohen (2, 3, where an insect uses highly refined sensory capabilities and specialized mouthparts to avoid certain food components such as toxins and to select other food components such as nutrient-rich tissues. Monarch larvae are well documented to use sensory and morphological adaptations to balance the intake of nutrients and either avoid or select portions of milkweed leaves (89–91).

An example of an artificial diet with a heterogeneous distribution of diet materials is presented here for the mile-a-minute weevil adult, *Rhinoncomimus latipes* Korotyaev (Curculionidae), a biological control agent of the weed *Persicaria perfoliata* (L.) H. Gross (Polygonaceae) (92) (**Figures 2L, M**). Further examples of heterogeneity in the organization of other components of natural foods include waxes, which tend to be on surfaces of plants and arthropods, and various other compounds such as plant secondary compounds (phenolics, flavonoids, terpenoids, etc.), toxins/feeding inhibitors such as cardenolides and latex, and structural components such as woody tissues, rinds, and arthropod cuticles.

#### 7.1.1 3D printing a diet that simulates a leaf

**Figure 3:**
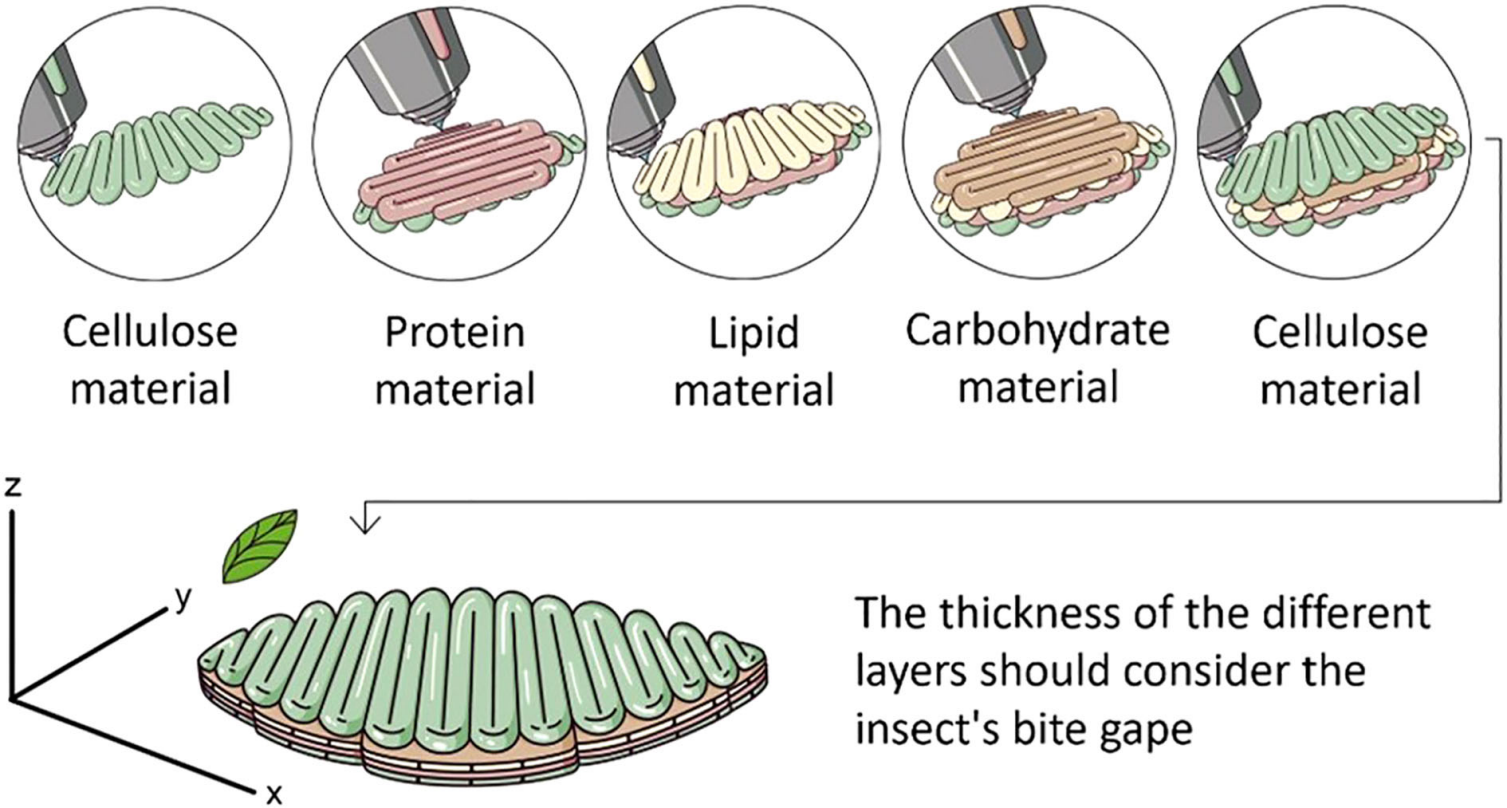
Depiction of 3D printing trough extrusion of an artificial diet mimicking a leaf. The idea is to create a diet with compartmentalized food components and a thickness appropriate for the bite gape of the mouth parts of the target insect. The example here shows a diet with layers of protein (e.g. yeast, casein), carbohydrate (e.g., sucrose) and lipid (e.g., cholesterol) rich materials, and surface materials such as cellulose.

Typical leaves are represented here by the cotton (*Gossypium* sp. (Malvales: Malvaceae)) leaf shown in **Figures 2A, B**, whose thickness is about 150 to 200 µM (0.150-0.200 mm). This agrees with the dimensions of the tea plant, *Camellia sinensis* L. (Ericales: Theaceae), as described by Zhu et al. (93). Using cotton or tea leaves as a model, a diet produced by 3D printing would consist of a layer of protein and carbohydrate-rich diet material such as wheat germ, soy flour, or other high protein/high starch components (**Figure 3**). The thickness of this layer would be about 150 to 200 µM, leaving at least 50-100 µM for layers of other materials such as lipid-rich liposomes, waxes, or other surface materials and fillers such as cellulose or chitosan membranes. Extrusion-based printing could be useful to manufacture such a diet as this technique allows using one or several materials to develop customized foods and the thickness of the filaments printed can be as little as 200 µm depending on the printer, the technique and the printing material (45). The idea is to produce a diet sheet that is similar to the thickness of a leaf or at least within the bite gape of the mouthparts of defoliators such as adult emerald ash borers (*A. planipennis*) (**Figure 2I**) whose gape is about 800 µM.

Once we know the nutritional and biochemical nature of natural foods that we are trying to mimic, and the 3-dimensional layout of these components, we can potentially simulate the composition and configuration of the components with “artificial materials”. To this end, liposomes (94, 95), variously described as spherical nanoparticles containing polar lipids (phospholipids), can be designed to contain various unstable nutrients (such as ascorbic acid and tocopherols) and secondary compounds (such as latex and cardenolides) which can be delivered in forms that are bioavailable stable in aqueous environments such as insect diets. Besides the composition and position, the particle size and other textural factors must be appropriate to mimic natural foods (96).

### 7.2 Fruit eaters

In this section we will focus on the cocoa pod borer, *Conopomorpha cramerella* (Snellen) (Lepidoptera: Gracillariidae), and tephritid fruit flies, to illustrate two systems of frugivorous insect pests whose rearing could be improved by designing 3D printed diets.

#### 7.2.1 Cocoa pod borer

**Figure 4:**
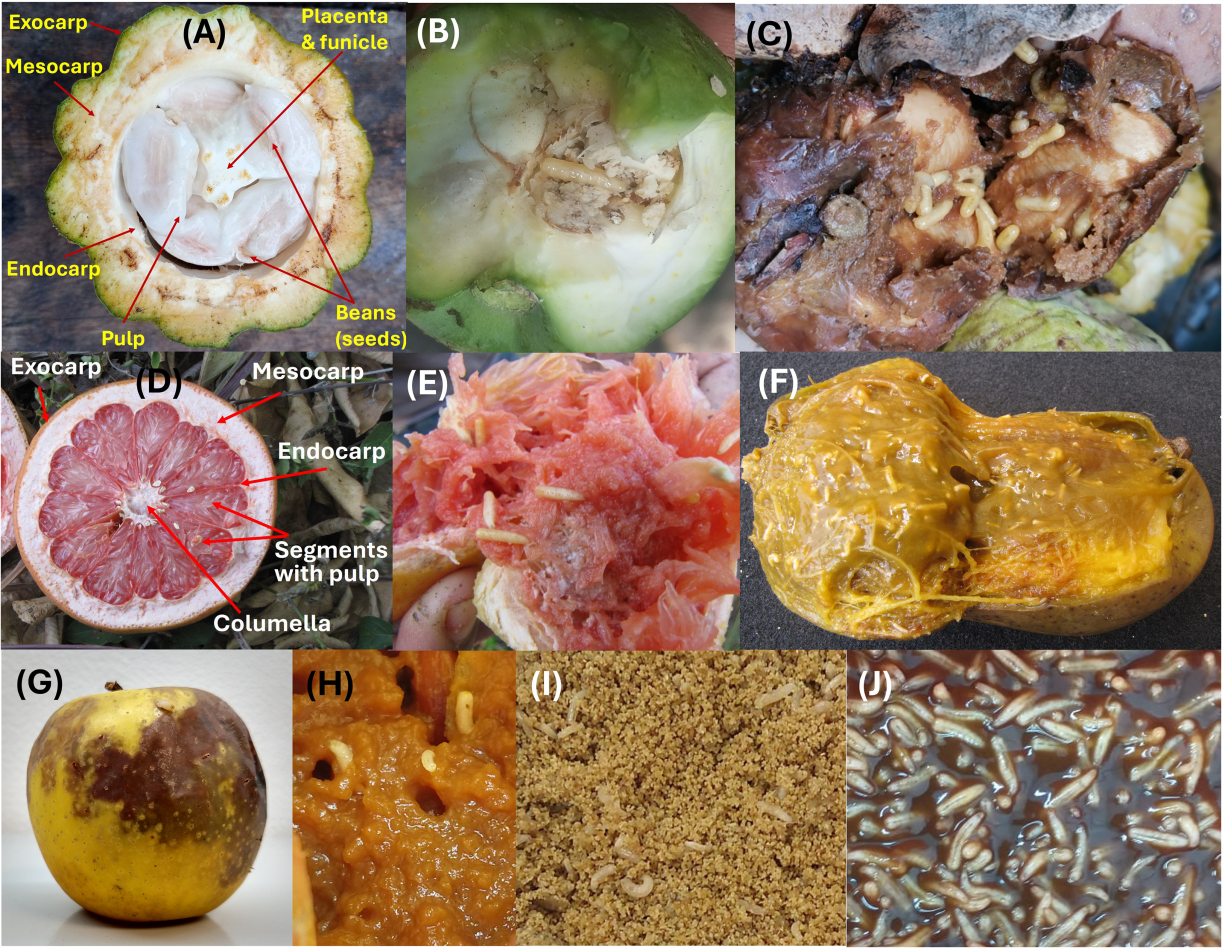
Examples of natural and artificial diets of frugivorous insects. **(A)** Cross section of a cocoa pod. **(B)** Yellow chapote (*Casimiroa greggii*) seed infested with third instar larvae of *Anastrepha ludens*. **(C)** White sapote (*Casimiroa edulis*) fruit infested with third instar larvae of *A*. *ludens*. **(D)** Cross section of a grapefruit (*Citrus paradisi*) cv ‘Ruby Red’. **(E)** Infested grapefruit with third instar larvae of *A*. *ludens*. **(F)** A mango (*Mangifera indica*) cv ‘Manila’ infested with third instar larvae of *A*. *ludens*. **(G, H)** Apple (*Malus domestica*) cv ‘Golden Delicious’ infested with third instar larvae of *A*. *ludens*. **(I, J)***A*. *ludens* second and third instar larvae developing on a corn cob powder and gel-based artificial diet, respectively. Photos credit: **(A)** José María Pascacio; **(B-H)** Emilio Acosta, Rafael Ortega and Erick Enciso; **(I, J)** Carlos Pascacio-Villafán.

This insect is a major pest of cocoa plants, *Theobroma cacao* L. (Malvaceae). Niogret et al. (97) developed artificial diets for *C. cramerella*, where the researchers included a partial analysis of the “husk” (pulp, funicle, and placenta, **Figure 4A**), a feeding target of the cocoa pod borer. The nutritional composition of the cocoa pod husk was used as a guide to improve the formulation of their artificial diets. For purposes here, the husk is considered the compartment targeted by *C. cramerella* as it invades and destroys cocoa pods.

The artificial diets designed to replace cocoa pods contain fresh or canned tomatoes, egg yolk, tapioca flour, lecithin granules, cellulose, brewer’s yeast, chickpea flour, red kidney bean flour along with a gelling agent (Phytagel or gellan gum), vitamins and water (97). The diet was mixed with a spatula and heated in spurts with a microwave to an unspecified temperature. Portions of diet were placed into 30 mL plastic cups, and individual *C. cramerella* eggs were assembled in the cup on the diet. This diet is representative of the convention of designing diets to be uniform in nutrient distribution. The uniformity of these diets raises questions of how well a more heterogeneous diet would suit the insect, which is adapted to feed on complex, non-uniform materials in the cocoa pod (**Figure 4A**). Before the advent of 3D food printing, non-uniformity of component distribution was difficult to achieve and would be considered a faulty practice because of introduction of errors due to unavailability of certain key components. This statement is based on the concepts put forth by Cohen (2) who pointed out that small organisms such as neonate insects might miss key nutrients such as lipids or vitamins if separated from bulkier components such as carbohydrate-rich materials in artificial diets. For this reason, instead of adding diet components to a homogenous mixture of ingredients as usual in artificial diet development, 3D printing technology could allow depositing units of certain materials in specific sections of the printed diet. The heterogeneity in the spatial distribution of diet components in a 3D printed diet could allow the insect to choose to consume specific materials found in identifiable compartments of the diet. Thus, the reared insect could self-select the combination of diet components that maximize fitness (98).

#### 7.2.2 Tephritid fruit flies

According to Zwölfer (99) tephritid fruit flies arose in the Tertiary, which means that they have been around for at least 25 million years. Most likely, their ancestral diet was decomposing organic matter, as related primitive groups (e.g., Uliididae) thrive on rotting plants/fruits (i.e., are saprophagous) (100–102). Currently, tephritids, particularly pestiferous ones, develop as larvae in living tissue, particularly the pulp of fruits and some vegetables (**Figures 4B–H**) (103). A notable exception is the Mexican fruit fly, *Anastrepha ludens* (Loew), a polyphagous species that is a pest of citrus and mango, whose larvae feed on the pulp of most host, but also on the seeds of one of its purported ancestral hosts (*Casimiroa greggii* (S. Watson) F. Chiang, (Rutaceae)) (104) (**Figure 4B**). Another wild host of *A. ludens* is White Sapote (*Casimiroa edulis* Llave et Lex) (**Figure 4C**). Considering our aims here, we will concentrate on a few of the highly polyphagous species that are mass reared for the application of the SIT (105), such as the Mediterranean (*Ceratitis capitata* Wiedemann), the Oriental (*Bactrocera dorsalis* (Hendel)), the Queensland (*Bactrocera tryoni* Froggatt), the South American (*Anastrepha fraterculus* (Wiedemann)) and Mexican (*A. ludens*) fruit flies, all of which commonly infest the fruits we will use here as models to develop our concept (i.e., citrus [*Citrus* x *sinensis* or sweet orange, and *C*. x *paradisi* or grapefruit, **Figures 4D, E**], mango [*Mangifera indica*] **Figure 3F**, peach [*Prunus persica* L. Batsch.] and apple [*Malus domestica* Borkh.] **Figures 4G, H**).

In nature, female flies lay their eggs into fruit/vegetables that are not fully ripe and devoid of damage by other insects or kin (103). This means that during oviposition the fruit tissue is hard and solid, with a relatively low nutrient content (106). Once the larvae eclose from the eggs (depending on the species, females lay clutches or single eggs), they start feeding on the pulp and a decomposition process ensues as microorganisms (bacteria, yeasts, fungi) grow fast and thrive in these types of media (107). As a result, and due to the natural ripening process of the fruit, the pulp softens and ends up transforming into a moist mush or slurry, almost liquid in some cases. The texture is watery in the cases of, for example, mango (**Figure 4F**) and peach. In the case of apples, the pulp with larvae feeding on it maintains its texture longer but becomes dark due to oxidation and eventually also transforms into a moist mush (**Figure 4H**). But independent of this, we note that, when ripe, the fruits we are using here as models (and many more), contain between 80-90% water, are rich in sugar and poor in protein and lipid content (**Table 1** and references therein). Also, they have a low content of dietary fiber and traces of micronutrients such as vitamins and minerals (**Table 1**).

**Table 1:** Nutritional content of four hosts (i.e., bitter orange, grapefruit, mango, peach, and apple) of the Mediterranean, Queensland, South American, and Mexican fruit flies.

Nutritional parameters	Unit	100 grams of fruit				
		Bitter orange	Grapefruit	Mango	Apple	Peach
Energy	Kcal	48	42	60	57	39
Water	g	87.6	88.5	81.1	85.8	88.9
Carbohydrates	g	10.9	10.66	15	13.6	9.54
Sugar	g	–	9.1	13.7	10	8.39
Protein	g	0.7	0.77	0.82	0.28	0.91
Total fat	g	0.2	0.14	0.38	0.15	0.25
Dietary fiber	g	0.3	1.6	1.6	2.4	1.5
Total Folate	µg	–	13	43	3	4
Niacin	mg	0	0.204	0.669	0.094	0.806
Pantothenic acid	mg	–	0.262	0.197	0.074	0.153
Pyridoxine (B6)	mg	–	0.053	0.119	0.051	0.025
Riboflavin	mg	0	0.031	0.038	0.026	0.031
Thiamin	mg	0	0.043	0.028	0.018	0.024
Vitamin C	mg	30	31.2	36.4	21.6	6.6
Vitamin A	IU	–	1150	1080	51	326
Vitamin E	mg	–	0.13	0.9	0.18	0.73
Potassium	mg	–	135	168	100	190
Calcium	mg	26	22	11	6	6
Copper	µg	–	32	0.111	0.03	0.068
Iron	mg	0.3	0.08	0.16	0.13	0.25
Magnesium	mg	–	9	10	5	9
Manganese	mg	–	0.022	0.063	0.035	0.061
Zinc	mg	–	0.07	0.09	0.04	0.17
α-Carotene	µg	–	3	9	0	0
β-Carotene	µg	–	686	640	25	162
Carotene	µg	1104	–	–	–	–
β-Cryptoxanthin	µg	–	6	10	12	67
Lycopene	µg	–	1419	1080	0	0
Phosphorous	mg	20	15	14	10	20
Sodium	mg	–	1	1	2	0

Data obtained from Kumar et al. (108), Abobatta (109), Waghmare et al. (110), Richa et al. (111) and USDA (112), for ripe fruit suitable for human consumption.

Considering the above, in their early stages of life, the larvae develop in an environment rich in secondary metabolites (many times deleterious) and low nutritional value (103, 106). As the fruit ripens, sugar content and moisture in the pulp increases considerably. In some cases, depending on the number of species and size of the population of the microorganisms that colonize the decomposing pulp (113), protein content can also increase (114). But importantly, protein for the larvae is mainly provided by bacteria in the gut (83, 115, 116). All this must be considered when developing artificial diets for these types of insects via 3D printing. The external color of fruit depends on the species and cultivar, but at least in the five species selected here as models, when they are unripe all are green, and then turn yellow/orange. The odor also varies greatly among species/cultivars, and this is important for female flies to locate hosts and oviposit into them.

As mentioned before, once the larvae start feeding in the undamaged pulp, microorganisms start to grow in this medium, and as a result the pulp turns from the original yellow (in all model fruits selected here), to brown (various tonalities thereof, from light to dark brown) (**Figures 4F, H**), which is also the color of some artificial diets (**Figures 4I, J**). An exceptional case is that of *A. ludens* feeding on the seeds of *C. greggii* (**Figure 4B**), as in this case the amount of water is much reduced, and the texture and chemical composition are very different when compared to rotting pulp.

When designing a 3D printed artificial diet for fruit fly larvae, it must be kept in mind that in nature these insects develop in changing/dynamic environments. Fruit pulp is hard/compact when the eggs are laid by females and the tiny first instar larvae eclose, but the second and third instar larvae complete their development in a moist and soft medium containing a mixture of macerated fruit pulp, microorganisms and water (**Figures 4F, H**). A 3D printed artificial diet should then, ideally, mimic these characteristics. Such a diet could be conceptualized as an all-in-one rearing system, serving the functions of an oviposition substrate for adult females, an egg incubation unit, and the environment in which the larvae will feed and coexists with conspecifics before metamorphosing into pupae, as in its host fruit in nature. The odor of fruit in a 3D printed diet could be included using volatile compounds from host fruits such as butyl hexanoate, hexyl hexanoate or α-farnesene present in apples; β-ocimene, β-pinene, and caryophyllene present in White Sapote; or D-limonene present in Sour Orange and Grapefruit (117). The internal anatomical and moisture characteristics of the 3D printed diet should allow the eggs to develop until they hatch without suffocating from lack of oxygen, drowning or dehydration. Because first instar larvae are likely to have different nutritional requirements than late instar larvae, it would be ideal to design a diet with a changing dietary environment that would fulfill the specific nutritional needs of the early and late instars. This could be achieved by using biopolymers (e.g., agar, alginate) to place units of carbohydrates, proteins, amino acids, beneficial microorganisms or any other dietary component in specific sections of the diet to which the larvae can have access as it grows and feeds. The original diet will change in many ways due to the activity of microorganisms, the feces of the larvae, and enzyme activities. It would also be potentially helpful to incorporate to the diet formulation pectin and hemicellulose or xylans, which are the natural connectors of cell walls (118). Also, the lignins would fit in as possible structural components that could be added artificially to simulate the natural arrangement of cells in the targeted fruits we are trying to simulate. We note that 3D-printed diets mimicking the morphological, textural and nutritional characteristics of host fruit could be valuable for tephritid SIT mass-rearing facilities seeking to maintain fly mother colonies in conditions as close as possible to nature to ease adverse effects of mass rearing (119, 120).

## 8 Challenges and limitations

3D food printing is an emerging technology whose application in the established field of artificial diet development and insect rearing pose challenges and limitations, many of which are common to all areas where this technology is applied (18, 121). Challenges that need to be overcome to render 3D printing artificial diets for insect rearing a reality include the economic investment in costly equipment and maintenance, the technical expertise required to operate the equipment and, importantly, the printing feasibility of nutritious materials (13, 44, 56).

As for equipment availability and costs for 3D diet printing research, several manufacturers already supply potentially suitable machinery (44). The cost of 3D food printers ranges from about $500 in the case of basic equipment designed to print only one ingredient to several thousand US dollars in the case of multi-ingredient equipment (44). The high initial cost of equipment is a barrier to the implementation of 3D food printing technology in the industrial-scale preparation of diets for mass rearing, an issue of scalability previously associated with this technology (43, 44, 56). However, it is expected that as 3D printing technology advances, the cost of equipment will decline (56). In fact, there are companies dedicated to the mass-production of 3D-printed food that have overcome the cost limitation by developing their own technology (38).

While the printing techniques are quite advanced for printing other kinds of materials, food materials still represent a considerable challenge due to the texture and rheological characteristics needed for printing complex structures when dealing with mixtures high in protein, carbohydrates, fats, and the combination of such macromolecules (40). The rheological properties of the matrix to be printed and their relationship to printability are of paramount importance when using an extrusion 3D printer, a topic that have been reviewed in detail elsewhere (33, 41, 43, 122–124). Also, a critical aspect for developing 3D-printed insect diets would be to accomplish complex textures, such as the ones in the porous structure of a fruit peel, so that the printed diet resembles as much as possiblethe insect’s natural food.

## 9 Future research and perspectives

The use of 3D food printing technology to develop artificial diets for insect rearing is a new field requiring an integrative approach from the perspectives of insect dietetics, nutrition, behavior and physiology, food engineering and chemistry. Research and development priorities should include addressing technological hurdles such as assessing the 3D printing feasibility of nutritious materials to achieve specific distribution of nutrients adapted to different insect species. Studying the rheological, textural, morphological and physicochemical properties of the printing materials will be needed as they influence the 3D printed structures (18, 43).

To achieve complex textures on 3D-printed diets, research should also be conducted on the structure of the natural diets that are to be simulated. In this regard, a recent study used X-ray microtomographic images of apple tissue as a digital model to print a cereal-based snack mimicking apple tissue microstructure (125). The use of software such as CURA (Ultimaker) or Tinkercad (Autodesk) to generate CAD models or G-codes should aid in designing diets with complex porous structures with controlled infills (41). The challenge of printing complex structures with a specific distribution/organization of diet materials for insects that micro-feed, could be researched by using coaxial extrusion-based techniques (126).

We note that the many advancements in the field of 3D printing can help us begin examining how this technology could be used to manufacture insect diets. For example, the existing models for the 3D printing of “beef” meat (37) could be useful for designing diets for laboratory-rearing necrophagous flies used in medicinal larval debridement therapy (127). The rearing diets of medicinal flies such as *Lucilia sericata* (Meigen) (Calliphoridae) include layers of red meat, ground beef and chicken liver (6), which could be possibly created by 3D printing using plant-based ingredients and specific chemical compounds to resemble the nutrient profile of meat (e.g., select proteins whose amino acid composition resemble the natural, original nutrients). 3D printing meat models (39, 40) could also be useful for rearing carnivorous insect species such as the New World Screwworm, *Cochliomyia hominivorax* (Coquerel) (Calliphoridae), a deadly pest of livestock and other warm-blooded animals including humans (128). 3D models also exist to print diets simulating the form of cherries, tomatoes, mangoes, oranges, lemons, strawberries and bananas (129, 130).

We highlight the potential applications of 4D printing using “smart materials” which, when exposed to external stimuli such as changes in pH, temperature, or light, can modulate color, shape, flavor, and/or nutritional content in a controlled manner (131). This technology could be key for the development of artificial diets that simulate the dynamic/changing environments of natural insect foods. Such diets could also help examine the responses of food components to the physical and chemical processes involved in insect feeding (e.g., stylet penetration, biting movements, and secretion of oral fluids).

The following steps outline a proposed framework for research and development of 3D-printed artificial diets. While the proposed steps are commonly applied in 3D food printing research (18, 132), they have been adapted to meet the requirements of insect rearing research:

1. Create a digital design of the diet to be printed. Digital models can be created by computer-aided design or by 3D scanning technology. A key aspect to consider when creating digital models is the spatial distribution of nutritional and non-nutritional components that the target insect encounters in its natural diet.
2. Selection of diet ingredients. A database of dietary ingredients for the target insect is required with information on their functionality (e.g., nutritional ingredients, texturizing and bulking agents, preservatives). If no information exists on the target insect, search for cases on related species on terms of diet and feeding habits.
3. Assess the rheological, textural, morphological and physicochemical properties of the printing materials. Ingredients or mixtures of ingredients should have consistencies that permit their flow through the printer nozzle and maintain their structure once they are printed.
4. Assess physicochemical characteristics of the 3D or 4D printed diet and perform feeding tests with the target insect.

## 10 Conclusion

With the currently available 3D printing technologies and equipment (e.g., extrusion 3D printers), and the associated software, it is possible to design customized artificial diets for insect rearing. However, much work lies ahead regarding the possible materials needed to balance both the nutritional needs of insects, and the technological feasibility for the materials to be printed. Despite the above, the wide variety of ingredients available for insect artificial diets including some food grade ingredients which have been reported as extrusion-printable materials, should ease the development of 3D-printed diet formulations. We hope this review will encourage new approaches to the development of insect artificial diets by using 3D food printing technology to simulate the non-homogeneous relationship or organization of diet components in natural insect foods.
